# Tuberculous Pericardial Effusion With Pleural Effusion in an Indian Female: A Rare Case

**DOI:** 10.7759/cureus.59546

**Published:** 2024-05-02

**Authors:** Sankalp Yadav

**Affiliations:** 1 Medicine, Shri Madan Lal Khurana Chest Clinic, New Delhi, IND

**Keywords:** diagnostic thoracentesis, pericardiocentesis, mycobacterium tuberculosis (mtb), pericardial effusion, tuberculous pleural effusion, tuberculosis

## Abstract

Tuberculosis can present at various extrapulmonary sites. However, even in endemic countries, concomitant involvement of different sites in the same patient is rarely reported. Further, tuberculous pericarditis represents a fraction of all tuberculosis infections and is an uncommon form of extrapulmonary tuberculosis. In underdeveloped nations, it is the most frequent cause of massive pericardial effusion. Additionally, it is the most common cause of constrictive pericarditis in adults, which has a high death rate and a poor prognosis. Furthermore, concomitant pleural effusion due to *Mycobacterium tuberculosis* is infrequently reported. Herein, a case of concomitant pericardial and left-sided pleural effusion in an Indian female is reported. She came with complaints of breathlessness, chest pain, night sweats, and loss of appetite. A diagnostic pleural thoracentesis and pericardiocentesis helped establish the diagnosis, and she was commenced on antituberculous treatment for 168 days.

## Introduction

Tuberculosis is a disease known for ages and is a substantial contributor to mortality and morbidity [1]. The disease is a result of infection due to the inhalation of infected aerosols [2]. Tuberculosis can occur in any organ of the body, but tuberculosis of the pericardium and pleura is relatively rare [3,4].

Tuberculous pericarditis represents 1-2% of all tuberculosis infections and is an uncommon form of extrapulmonary tuberculosis. In endemic nations, it is the most frequent cause of extensive pericardial effusion [3]. Additionally, it is the most frequent cause of constrictive pericarditis in adults, which has a significant mortality rate and a poor prognosis [5].

Tubercular pleural effusion is due to the presence of *Mycobacterium tuberculosis* in the pleural space. Chronic, severe buildup of inflammatory cells and fluid in the pleural area is its defining feature [6]. Pleural involvement in tuberculosis varies from 3-5% in non-endemic areas to up to 30% in endemic ones [4].

A case of simultaneous involvement of pleural and pericardial effusion in an Indian female is presented here. The diagnosis was challenging due to the invasiveness of the diagnostic techniques.

## Case presentation

A 52-year-old Indian female belonging to a low socio-economic background reported complaints of breathlessness and chest pain for one month. It was associated with night sweats and a loss of appetite for 20 days. She was well 30 days ago when she developed chest pain localized to the left side with dyspnea. Initially, the dyspnea was grade 1 on the modified Medical Research Council dyspnea scale, but it progressed to grade 3 in the last 15 days when doing routine household work. However, there was no cough, fever, or recorded loss of weight. She was a housewife with no history of tuberculosis or surgical intervention in the past. However, she was a known hypertensive (taking tablet enalapril 5 mg once daily for five years) and diabetic with diabetes mellitus type 2 (on metformin 500 mg twice a day for 10 years).

Upon general assessment, the female with an ectomorphic build was in hemodynamic equilibrium. No icterus, pallor, clubbing, cyanosis, edema, or lymphadenopathy were present. The diminished movement on the left hemithorax during the systemic examination was noteworthy. On the left, there was also a decrease in tactile vocal fremitus and breath sounds. There was significant egophony in the lower left lobe. The entire left lung field was heard with noisy crepitations on auscultation. On room air, the peripheral oxygen saturation was 96%, but the respiratory rate was normal and there were no symptoms of respiratory distress at rest. Further, it fell to 92% with a respiratory rate of 34/min on exertion. Her blood pressure was 120/80 mm Hg and her pulse was 80/min. The cardiovascular examination was remarkable for muffled heart sounds. The rest of the systemic assessment went forward without any notable findings.

**Figure 1:**
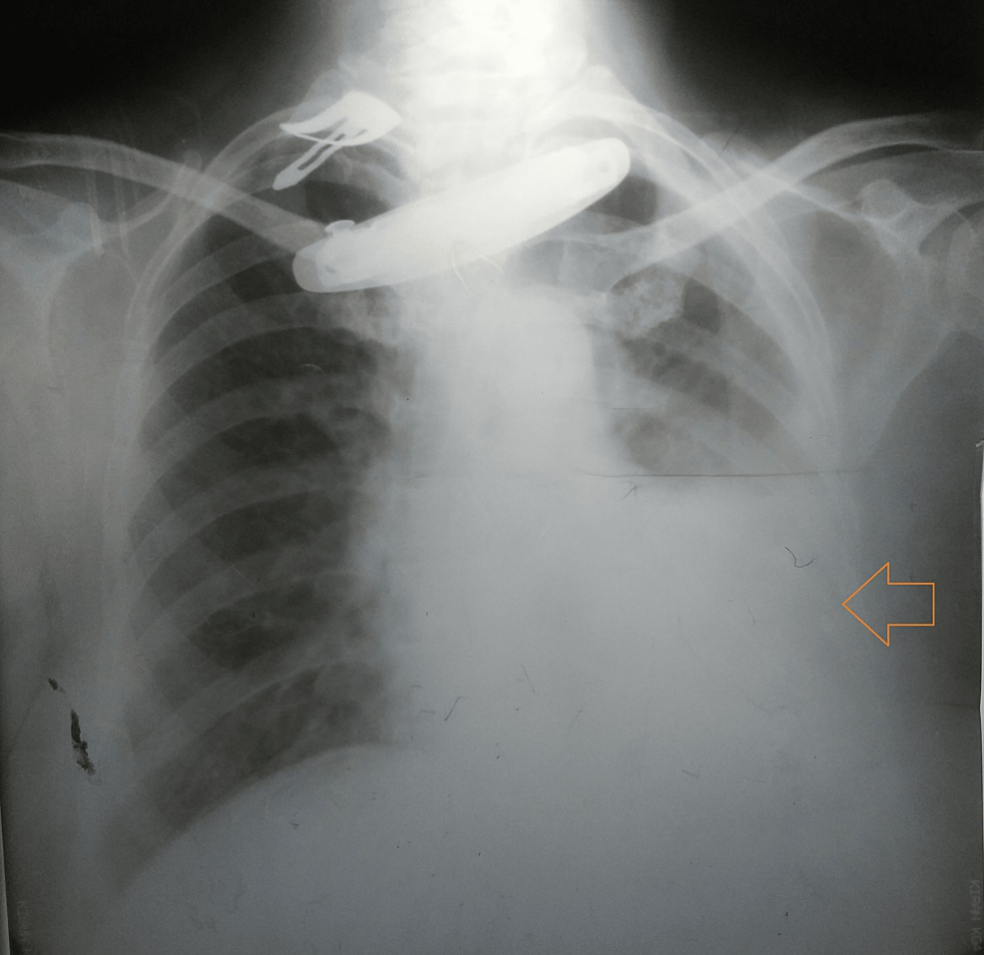
Chest radiograph (PA view) suggesting cardiomegaly with left pleural effusion PA: Posteroanterior

A chest radiograph was suggestive of a cardiomegaly with left pleural effusion (Figure 1).

An electrocardiogram was unremarkable. An ultrasound of the chest was suggestive of a left-sided pleural effusion with pericardial effusion.

**Figure 2:**
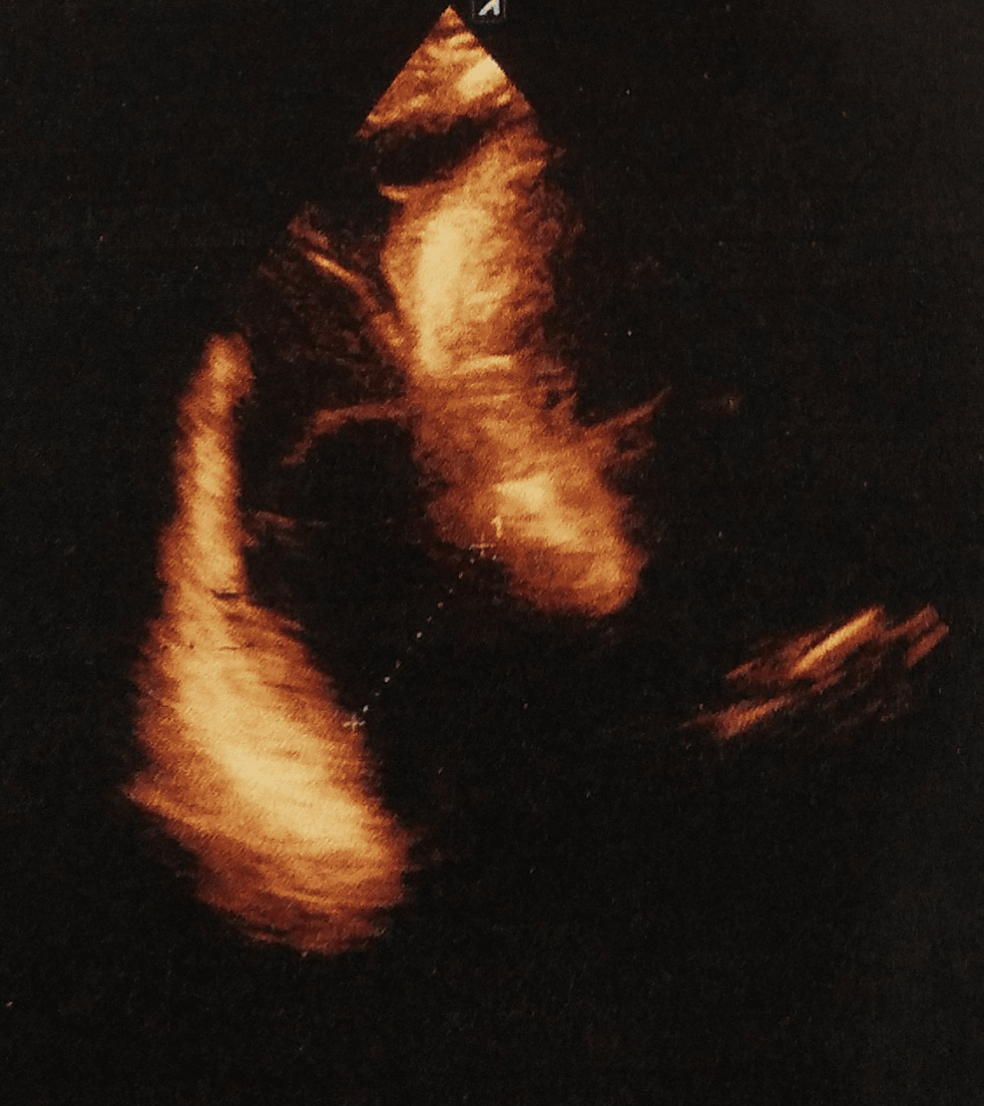
Color Doppler echocardiography showing massive pericardial effusion

A color Doppler echocardiography was remarkable for mild concentric left ventricular hypertrophy. There was a massive pericardial effusion with a posterior depth of 3.8 cm and a lateral depth of 3.2 cm. There was a diastolic relaxation abnormality with trace tricuspid regurgitation. The left ventricular ejection fraction was 60% (Figure 2).

Her lab panel was remarkable for a raised erythrocyte sedimentation rate of 67 mm per hour with a Mantoux test positive with 15 x 15 mm induration. However, an induced fluorescent sputum microscopy for *Mycobacterium tuberculosis* and a cartridge-based nucleic acid amplification test of the sputum were negative. Her glycated hemoglobin (HbA1c) was 6.5%.

Due to the endemicity of the disease, a provisional diagnosis of tuberculosis with differentials as malignancy was made. She was referred to a tertiary care center, where a diagnostic thoracentesis was done, and the results are shown in Table 1.

**Table 1:** Results of diagnostic thoracentesis ADA: Adenosine deaminase; CBNAAT: Cartridge-based nucleic acid amplification test; MTB: *Mycobacterium tuberculosis*; LDH: Lactate dehydrogenase

Test	Result	Reference range
Physical appearance	Straw colored	Colorless
Protein	5.8	1-2 g/dL
Glucose	29 mg/dL	74-106 mg/dL
pH	7.6	7.60-7.64
Cells	90% lymphocytes	75% macrophages
ADA	59.9	<30 U/L
CBNAAT	Negative for MTB	-
Culture	Sterile	-
Pleural fluid protein/serum protein ratio	0.80	-
Pleural fluid LDH	>1,500 U/L	>1,000 U/L
Mesothelial cells	Not seen	-

Further, she was also referred to another tertiary care center for pericardiocentesis, and the results are tabulated in Table 2.

**Table 2:** Results of pericardiocentesis ADA: Adenosine deaminase; CBNAAT: Cartridge-based nucleic acid amplification test; MTB: *Mycobacterium tuberculosis*

Test	Result	Reference range
Physical appearance	Cloudy	Colorless
Protein	5.2	1-2 g/dL
Glucose	33 mg/dL	74-106 mg/dL
Cells	91% lymphocytes	75% macrophages
ADA	62	<30 U/L
CBNAAT	Negative for MTB	-
Culture	Sterile	-
Gram stain	Negative	-

Hence, based on the reports and after ruling out other differentials like rheumatoid pleurisy (rheumatoid factor negative), empyema (polymorphonuclear neutrophils predominance not seen), and lupus pleuritis (polymorphonuclear neutrophils predominance not seen with antinuclear antibody negative), a final diagnosis of tuberculous pericardial effusion with left-sided pleural effusion was made. She was initiated on antituberculous chemotherapy for the first two months as an intensive phase with fixed-drug combinations of four drugs (rifampicin, ethambutol, pyrazinamide, isoniazid), followed by a continuation phase of three drugs (isoniazid, rifampicin, and ethambutol) for 112 days. She was advised to consult for sugar control and hypertension in a nearby medicine outpatient department. A follow-up chest radiograph was inaccessible as the patient was transferred out of the state due to personal reasons. However, her treatment outcome in the national tuberculosis portal was marked as treatment complete.

## Discussion

Tuberculosis is commonly reported in high-burden countries. The disease is a remarkable threat to the healthcare system, with about 1.3 million people dying due to it in 2022 [7]. Extrapulmonary tuberculosis is reported in various organs, but concomitant involvement of the pleura and pericardium is rare.

Pericardial tuberculosis is an uncommon paucibacillary manifestation of extrapulmonary tuberculosis. With an estimated 1-4% incidence of pericarditis, it typically arises from retrograde spread from peritracheal, peribronchial, or mediastinal lymph nodes, or hematogenous spread from the lung, spine, sternum, or during miliary infection [8]. Further, it has a notable impact on cardiovascular death and disability [9].

Due to its paucibacillary nature, tuberculous pleural effusion is difficult to diagnose with only laboratory tests and requires invasive procedures like thoracentesis. Moreover, the optimal diagnostic strategy for suspected tuberculous pleuritis is still debatable [10]. There is a 10% yield for pleural fluid smears, while 25-85% is the yield for pleural fluid cultures [11]. Diagnostic yields for culture or granulomas from pleural biopsy and its histopathology range from 55-93%. Thoracentesis combined with a closed pleural biopsy provides 95% sensitivity, which is comparable to thoracoscopy. However, all these diagnostic modalities take longer to produce data, which makes acute workup challenging [10]. Additionally, tests like cartridge-based nucleic acid amplification tests or Xpert MTB/RIF Ultra could be used for such samples with a low bacterial load [12]. Nevertheless, a strong suspicion due to clinical features of tuberculosis in an endemic country with an exudative picture oF the diagnostic thoracentesis and pericardiocentesis is sufficient to initiate antituberculous chemotherapy.

Clinical features of both pericardial effusion and pleural effusion are non-specific like dyspnea, cough with or without expectoration, chest pain, night sweats, orthopnea, weight loss, and lower limb edema [8]. Additionally, in pericardial effusion, the commonest signs are cardiomegaly, pericardial friction and tachycardia with a paradoxical pulse, hepatomegaly, jugular stasis, and pleural effusion [10].

Diagnostic radiographs are indicative of cardiomegaly in more than 90% of patients [10]. Almost all cases of tuberculous pericardial effusion have aberrant electrocardiograms, most commonly in the form of non-specific ST-wave anomalies. An effective and non-invasive technique for detecting cardiac tamponade and pericardial effusion is an echocardiogram. Though not unique to a tuberculous etiology, the development of an effusion in the visceral pericardium with fibrinated fibers is indicative [13].

Advanced radiometric techniques like computed tomography of the chest show typical changes in the mediastinal lymph nodes in almost all of the cases [14]. In addition, the degree of pericardial involvement can be precisely defined and quantified. As far as possible, the tuberculous etiology of pericarditis should be determined by carefully looking for acid-fast bacilli in the pericardial fluid, lymph nodes, and sputum [13]. However, exudative pleural and pericardial fluids on thoracentesis and pericardiocentesis are diagnostic in these paucibacillary samples.

The management is essentially medical, with antituberculous chemotherapy for 168 days with a provision to extend the treatment based on clinical assessment [15]. The use of therapeutic thoracentesis is debatable. However, few studies have demonstrated its effectiveness in moderate-to-severe symptomatic cases. A chest tube insertion is often the initial procedure for massive pleural effusions. Also, the use of intrapleural tissue plasminogen activator/deoxyribonuclease (tPA/DNase) therapy in tuberculous pleural effusions has been reported to improve fluid drainage and lessen residual pleural thickening [16].

Adjunct corticosteroid therapy lowers the incidence of patients developing constrictive pericarditis and the hospitalization rate, but it raises the risk of malignancy in patients who are HIV positive. Constrictive pericarditis is thought to be less common when routine pericardiocentesis is performed with prolonged drainage, according to certain studies [3].

A case similar to the present case was reported by Abdelghani et al. in a 59-year-old male [17]. The present case shares similarities with theirs in the simultaneous involvement of both the pleura and pericardium. But the present case differs in unilateral pleural effusion compared to bilateral in theirs [17]. Further, the present case is in a female with no contact with tuberculosis in the family, and there was no pericardial mass.

## Conclusions

A case of an Indian female with concomitant pleural and pericardial effusion due to tuberculosis is presented. This case underlines the importance of having a clinical suspicion of the involvement of multiple sites with tuberculosis in endemic settings. A timely diagnosis and management are imperative as delays could result in chronic constrictive pericarditis or the development of drug resistance. It is absolutely essential to perform diagnostic thoracentesis and pericardiocentesis to establish a definite diagnosis in paucibacillary tuberculosis cases.
